# Soilless Cultivation: Precise Nutrient Provision and Growth Environment Regulation Under Different Substrates

**DOI:** 10.3390/plants14142203

**Published:** 2025-07-16

**Authors:** Arezigu Tuxun, Yue Xiang, Yang Shao, Jung Eek Son, Mina Yamada, Satoshi Yamada, Kotaro Tagawa, Bateer Baiyin, Qichang Yang

**Affiliations:** 1 Research Center for Smart Horticulture Engineering, Institute of Urban Agriculture, Chinese Academy of Agricultural Sciences, Chengdu National Agricultural Science and Technology Center, Chengdu 610213, China; 2 Yazhouwan National Laboratory, Sanya 572025, China; 3 Department of Agriculture, Forestry and Bioresources, Seoul National University, Seoul 08826, Republic of Korea; 4 Faculty of Agriculture, Tottori University, Tottori 680-8553, Japan

**Keywords:** soilless cultivation, solid substrate, nutrient solution, rhizosphere microorganisms, smart agriculture

## Abstract

Soilless cultivation technology is a key means of overcoming traditional agricultural resource limits, providing an important path to efficient and sustainable modern agriculture by precisely regulating crop rhizospheric environments. This paper systematically reviews the technical system of soilless cultivation, nutrient solution management strategies, the interaction mechanism of rhizosphere microorganisms, and future development directions, aiming to reveal its technical advantages and innovation potential. This review shows that solid and non-solid substrate cultivation improves resource utilization efficiency and yield, but substrate sustainability and technical cost need urgent attention. The dynamic regulation of nutrient solution and intelligent management can significantly enhance nutrient absorption efficiency. Rhizosphere microorganisms directly regulate crop health through nitrogen fixation, phosphorus solubilization, and pathogen antagonism. However, the community structure and functional stability of rhizosphere microorganisms in organic systems are prone to imbalance, requiring targeted optimization via synthetic biology methods. Future research should focus on the development of environmentally friendly substrates, the construction of intelligent environmental control systems, and microbiome engineering to promote the expansion of soilless cultivation towards low-carbon, precise, and spatial directions. This paper systematically references the theoretical improvements and practical innovations in soilless cultivation technology, facilitating its large-scale application in food security, ecological protection, and resource recycling.

## 1. Introduction

With the global population growth [1], the sharp reduction in cultivated land resources, and the intensification of climate change [2], traditional soil-based agriculture is facing multiple challenges, including soil degradation [3], water shortages, and environmental pollution [4]. In this context, soilless cultivation technology is gradually becoming the key direction to promote the transformation of modern agriculture because it can break through geographical and ecological constraints and achieve efficient use of resources [5] due to its potential to overcome geographical and ecological limitations and achieve efficient resource utilization. It creates optimal growth conditions for crops by artificially regulating the root environment [6], such as nutrient supply [7], gas exchange [8], and microbial communities [9]. This not only significantly improves the yield and quality per unit area [10] but also expands agricultural production space through models such as vertical farming, cultivation in desert areas, and urban farms [11]. According to the prediction of the Food and Agriculture Organization of the United Nations, the global food demand will increase by 60% by 2050 [12]. As an important part of precision agriculture, soilless cultivation is a key technology to ensure food security and relieve ecological pressure [13].

In recent years, the technical system of soilless cultivation has become increasingly mature, and its research scope has expanded from single-facility optimization to multi-disciplinary cross-fields [5]. The technological innovations in solid and non-solid substrate cultivation have significantly improved the controllability of the root environment and the production stability [14]. Refining dynamic management strategies for nutrient solutions, such as conductivity regulation [15] and the application of biostimulants [16], has further optimized the nutrient absorption efficiency of crops. Meanwhile, the analysis of the interaction mechanism between rhizosphere microorganisms and plants has revealed the key role of the microbiome in enhancing stress resistance, antagonizing pathogens, and promoting nutrient cycling, providing a new perspective for the ecological design of soilless cultivation systems [17,18]. However, there are still significant challenges in this field: the non-degradability and recycling difficulties of traditional substrates restrict their sustainability [19]; the high cost of automation systems hinders the popularization of the technology [20]; the complex interaction mechanism of microbial communities has not been fully clarified, and it is prone to cause microbial imbalance, especially in organic soilless cultivation [21].

Current research urgently needs to break the existing bottlenecks through technological innovation and interdisciplinary integration. For example, developing environmentally friendly substrates based on agricultural waste, constructing intelligent environmental control systems, and using synthetic biology (methods of rebuilding biological systems by engineering design) to design functional microbial communities in a targeted manner are all important paths to achieve the green transformation of soilless cultivation. Therefore, in order to understand the main forms of soilless culture, the relationship between soilless culture and microorganisms, and the application fields, “soilless culture, solid matrix, non-solid matrix cultivation, hydroponics, aeroponics, gel culture, nutrient solution, soilless culture and microorganisms” were used as keywords. According to Web of Science, PubMed, and other databases, relevant literature from the past five years was mainly retrieved. This paper systematically reviews the technical progress of soilless culture, analyzes its core advantages and limitations (Table 1), and looks forward to the future research direction, aiming to provide a theoretical reference for the academic community and promote the development of soilless culture technology in the direction of high efficiency, low carbon, and sustainability.

## 2. Solid Substrate Soilless Cultivation

**Figure 1:**
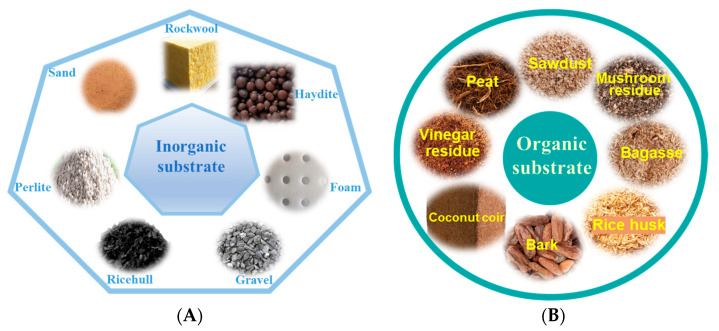
Solid substrate of soilless cultivation. (**A**) Inorganic solid substrate. (**B**) Organic solid substrate.

Solid substrate cultivation uses non-soil materials, supplies the water and nutrients required for crop growth and development by irrigating nutrient solutions. Compared to hydroponics, substrate cultivation requires simpler facilities and lower costs (Table 2). Since the substrate has a buffering effect, environmental changes such as those in nutrients, moisture, and temperature are more gradual. The techniques are quite like traditional soil and are easy to master. Substrate cultivation production requires a large amount of substrate material. Based on the substrate materials used for cultivation, it can be divided into inorganic and organic substrate cultivation [22]. (See Figure 1).

### 2.1. Inorganic Solid Substrate Cultivation

Inorganic substrates are widely used as a primary cultivation medium. Commonly used ones include rockwool [23], sand [24], gravel [25], ceramsite [26], vermiculite [27], and perlite [28,29]. Their physical and chemical properties are relatively stable, but their fertilizer-holding capacity is poor. Soilless cultivation using rockwool as the substrate is called rockwool cultivation. Rockwool is a solid substrate formed by melting multiple rocks, spraying them into filaments, and fusing them after cooling. It is loose, porous, and moldable. Rockwool for soilless cultivation first appeared in Denmark in 1963, and the Netherlands applied it to soilless cultivation in 1970. Rockwool is lightweight, resistant to rotting and decomposition, and has good air permeability. Currently, the use of rockwool in soilless cultivation ranks first globally.

As a common technology in soilless cultivation, although rockwool cultivation has advantages such as a controllable root environment and high water–fertilizer utilization efficiency, its disadvantages cannot be ignored. After use, rockwool may retain roots, salts, and pathogens. Recycling and reusing it requires high-temperature disinfection or chemical treatment, which is costly and technically complex [30,31]. Qaryouti et al. found that date palm waste can be an environmentally friendly alternative to rockwool in soilless cultivation systems. Although rockwool performed better in plant growth parameters, the results showed no significant difference in the total yield and quality of sweet peppers between the two substrates [32]. An et al. compared the effects of two soilless cultivation substrates, rockwool and coconut coir, on the growth and development of greenhouse strawberries. They found that strawberries grown in rockwool performed better in reproductive growth and fruit yield, while strawberries in coconut coir medium were more vigorous in vegetative growth [33]. In the soilless cultivation of tomatoes in greenhouses, no significant difference was noted in the effects of rockwool, reused rockwool, and coconut coir medium on plant growth and development [15]. The yield and quality of tomatoes are more affected by environmental conditions and water management strategies than by the substrate. Reused rockwool performs comparably to new rockwool, and it has the advantages of economic feasibility and environmental sustainability, which can reduce resource waste.

Sand is widely available from rivers, seas, lakes, and deserts. Coupled with its low cost, it was one of the earliest substrate materials used in soilless cultivation. However, its disadvantages include a large bulk density, poor water-holding capacity, and significant chemical composition and quality differences depending on the source (Table 3) (based on Wallach, R., 2019 [34]). Sand culture is an open-type soilless cultivation system that uses sand as the substrate. Sand culture can be regarded as gravel culture. Still, its substrate’s particle size is smaller than gravel cultures, and its water-retention capacity is higher than gravel cultures. The physical properties vary greatly with different particle-size compositions of sand, determining the cultivation effect.

The sand particle size and phosphorus content significantly affect the spore production and mycorrhizal colonization efficiency of *Rhizophagus irregularis*. Excessively fine sand particles (106–300 μm) can lead to nutrient saturation due to overly strong water-retention capacity, inhibiting spore formation [35]. Giroto et al. showed that adding zinc to urea-formaldehyde nanocomposites can significantly improve sand culture’s nitrogen and zinc fertilizer efficiency [36]. It effectively reduces the volatilization loss of nitrogen. The loss rate of traditional urea is as high as 50%, and it synchronously optimizes the release of zinc. The composite material significantly promotes the root development of maize, such as increasing root length and surface area, thereby enhancing the nutrient absorption capacity. The soilless cultivation method using gravel as the cultivation substrate is called gravel culture. It has a poor ability to retain water and nutrients [37], but it exhibits good ventilation and drainage performance. Gravel played an important role in the early stage of soilless cultivation. In 1972, a research report indicated that the nitrate reductase activity of soybeans in gravel culture was significantly higher than that of soybeans grown in soil, suggesting that under normal field conditions, soybeans may not fully realize their nitrate utilization potential [38].

As a porous material, perlite’s water-absorption property is widely used in multiple fields. Research shows that the water absorption of perlite can reach 2–3 times its mass, which is mainly attributed to its unique pore structure and hydrophilic surface characteristics [39]. Some scholars have found that the use of perlite significantly affects the emission rate and composition of root exudates of lettuce [40]. It also increases the root exudate carbon emission rate and carbohydrate emission rate of roots, especially in the early growth stage. In addition, perlite affects roots’ growth and metabolic activities through its physical and chemical properties, such as particle size, mechanical impedance, adsorption capacity, and air permeability. The research also found that the interaction between light quality and perlite significantly impacts the emission of root exudates, especially under red-light conditions. These results indicate that perlite not only directly affects the emission of root exudates but also indirectly affects the structure and function of the rhizosphere microbial community through its synergistic effect with light quality, thus impacting plant growth and health.

### 2.2. Organic Solid Substrate Cultivation

Organic substrates mainly refer to cultivation substrates composed of organic biological residues containing carbon and hydrogen and their derivatives, such as peat [41], vinegar residue [42], coconut coir [43], bark [44], sawdust [45], and mushroom residue [46]. Organic substrates’ chemical properties are less stable than inorganic ones. They usually have a relatively high cation exchange capacity and relatively strong abilities to retain fertilizers and water [45]. Peat is one of the best organic substrates for soilless cultivation worldwide. Although its effect is limited when used alone, when mixed with other substrates, it can significantly improve the growth performance of banana plants, including increasing the number of leaves, stem diameter, leaf length, chlorophyll content, and enhancing the absorption of nitrogen, phosphorus, and potassium. Mixing peat with vermicompost can also improve the substrate’s aeration, water-holding capacity, and nutrient supply capacity, promoting root development and nutrient absorption. Moreover, this combination may enhance chlorophyll synthesis and mineral absorption efficiency through vermicompost’s hormonal and enzymatic activities. Peat is not the optimal treatment for banana plant cultivation; however, it is an efficient auxiliary cultivation substrate when rationally proportioned with organic materials such as vermicompost [47]. When studying the effects of peat, coconut coir, and bark on blueberry cultivation, Kingston et al. found that a high proportion of peat or coconut coir substrate significantly promoted blueberry growth, increased its dry weight, and nutrient absorption efficiency. In contrast, a high proportion of bark substrate led to poor blueberry growth due to its poor water-holding capacity, low porosity, and insufficient nutrient supply, especially when its proportion exceeded 30%. Peat and coconut coir perform better in terms of nutrient availability, especially in the supply of nitrogen, phosphorus, and potassium [48].

In soilless cultivation, organic substrates’ physical and chemical properties significantly affect lettuce growth. Nerlich et al. compared the performance of sawdust, peat, hemp fiber, and rockwool. They found that peat, with its good water-holding capacity, moderate decomposition rate, and low pH value, could support high lettuce yields and medium phenolic acid content, making it the best alternative to rockwool [45]. Hemp fiber severely inhibited lettuce growth and led to extremely low yields due to rapid decomposition, which caused oxygen deficiency and nitrogen fixation. Although the yield of lettuce grown with sawdust was relatively low, optimizing the nutrient solution could improve its performance. In addition, organic substrates can be used as farmland fertilizers, reducing waste and enhancing sustainability. Organic substrates are environmentally friendly and can potentially balance crop yield and quality. Lu et al. used straw to replace wood to cultivate *Hericium erinaceus*. They successfully screened an efficient and environmentally friendly substrate formula (16.3% rice straw, 59.7% corncob, 20.0% wheat bran, 2.0% gypsum, 1.0% sucrose, and 1.0% superphosphate). This formula performed excellently regarding mycelial growth rate, laccase, cellulase, and neutral xylanase activities. It significantly improved the biological efficiency of *Hericium erinaceus* (89.14%) and shortened the fruiting cycle (7–9 days). This provides a scientific basis for the large-scale cultivation and quality improvement of *Hericium erinaceus*, while reducing the dependence on wood resources and environmental pollution [49].

## 3. Non-Solid Substrate Cultivation

### 3.1. Hydroponics

Hydroponics is the main form of soilless cultivation. It uses nutrient solution as the cultivation medium, which can be independently adjusted according to cultivated crop type, growth stage, cultivation season, and quality requirements. The nutrient solution can provide all the nutrients needed for plant growth and can be precisely controlled, making it a high-tech and advanced modern agricultural cultivation method. Based on differences in the depth of the nutrient solution layer, facility structure, and management measures for oxygen and nutrient solution supply, hydroponics can be divided into two major types: First, the deep-flow hydroponics technology, sometimes also called deep-water hydroponics technology [50]. In this method, the nutrient solution layer is relatively deep. Plants are suspended above the surface of the nutrient solution by a planting board or a planting net frame, from which the roots extend into the nutrient solution for growth. Second, the nutrient film technique is also known as shallow-water hydroponics [51]. Here, the nutrient solution layer is relatively shallow. The plants and their planting pots are placed directly at the bottom of the planting trough. The roots grow at the bottom of the trough, most exposed to the humid air, while the shallow-layer nutrient solution flows at the bottom. Other hydroponic forms have emerged. To fully utilize the advantages of hydroponic technology, many countries and regions have developed various forms of hydroponics tailored to local conditions. Some are improvements based on deep-flow or nutrient film hydroponics, some are combinations of the two, and others are designed to meet the needs of home hydroponics. For example, floating capillary hydroponics, deep-water floating cultivation [52], multi-functional trough hydroponics, vertical leafy vegetable hydroponics, and small-scale hydroponics [53].

Deep-flow hydroponics has a deep nutrient solution layer. Kaur et al. conducted research using deep-flow hydroponics technology. By using sensors to adjust parameters such as temperature, humidity, pH value, and nutrient solution concentration in real-time, they found that the automated system significantly outperformed the non-automated system that relied on manual adjustment in terms of plant growth indicators [50]. Although plants in both systems met their nutritional requirements, the automated system could maintain optimal environmental conditions more steadily. Despite the automated system’s high initial cost and energy consumption, its advantages in increasing yield and resource efficiency are obvious, making it suitable for large-scale applications. The non-automated system is more suitable for small-scale scenarios. Future research can combine machine learning for optimized control and explore the effect of carbon dioxide supplementation on plant growth [50].

The nutrient film technique is a relatively simple hydroponic method where plants are grown in a shallow, flowing nutrient solution. The investment in facilities is relatively low. Chowdhury et al. evaluated the performance of different hydroponic systems for producing organic lettuce using liquid organic fertilizers in a controlled environment [53]. They found that substrate-based systems (such as Dutch buckets (DB) and regular plastic containers (RPC)) significantly outperformed liquid culture systems. Regarding growth parameters such as stem width, number of leaves, leaf area, fresh weight, and dry weight, lettuce in the substrate systems was 15–60% higher than in the liquid systems. The root development was also better, possibly due to the microbial activity in the substrate promoting nutrient mineralization. Although the growth of lettuce in the nutrient film technique system was the weakest, the mineral content in its leaves was comparable to or even higher than that in the substrate-based systems. This suggests that system design influences nutrient absorption efficiency.

### 3.2. Aeroponics

Aeroponics refers to a cultivation method where the roots of crops are suspended and grow in a closed, light-impermeable container. Special equipment atomizes the nutrient solution into a mist and intermittently sprays onto the crop roots. However, some researchers have conducted light treatment on the roots of medicinal plants *Artemisia annua* and *Hypericum perforatum* in aeroponics. This can significantly promote the accumulation of secondary metabolites in the above-ground parts of the plants through systemic signal transduction without significantly inhibiting plant growth.

Aeroponics can be divided into semi- and full-aeroponics, depending on whether the roots are briefly immersed in the nutrient solution layer. The greatest advantage of aeroponics is that it effectively resolves the contradiction between oxygen supply and nutrient solution supply for hydroponic roots without using additional energy. However, the initial investment in aeroponic facilities is relatively large. Also, due to the poor buffering capacity of the rhizosphere environment of aeroponically grown crops, aeroponics has relatively strict requirements for the temperature and humidity of the growth environment [54,55].

Farqani et al. used an aeroponic system to supply nutrient solution to apple roots and set up three treatments with pH values of 5.5, 6.5, and 8.0, respectively [56]. A higher pH value led to a decrease in the average width of the roots, and structural parameters such as the length-to-width ratio of the roots and the network width-to-depth ratio showed significant differences under different pH values. In addition, although the differences in leaf nutrient concentrations among different rootstocks were not significant, the solution pH value significantly impacted the concentrations of phosphorus, calcium, and manganese. This study explored the effect of pH on apple root structure in an aeroponic environment, providing a new perspective for understanding the growth behavior of apple rootstocks under different soil pH conditions and potentially offering references for orchard soil management and irrigation water quality optimization.

Jamshidi et al.’s research showed that the ultrasonic frequency and atomization time significantly impacted the EC and pH values of the nutrient solution and tomato yield [57]. The tomato yield was the best at a frequency of 50 kHz and an atomization time of 15 min. Nevertheless, further research is still needed to determine the optimal combination of ultrasonic frequency and atomization time to optimize the performance of the aeroponic cultivation system. The high energy consumption of ultrasonic atomizers may limit their application in large-scale agricultural production (Table 4).

Weingarten et al. systematically evaluated the propagation efficiency of *Cannabis sativa* L. cuttings by comparing soilless cultivation methods such as aeroponics, rockwool, and phenolic foam, combined with optimization experiments on spray intervals and nutrient solution concentrations. The study found that aeroponics performed excellently in root development, plant height, and biomass accumulation, especially under continuous spraying and high nutrient solution concentration (EC 1.4 dS/m). Significant differences were observed in the responses of different varieties to cultivation conditions, and targeted optimization strategies were needed [58]. Although aeroponics relies on electricity and has a high initial cost, its characteristics of efficient resource utilization and reduced substrate waste make it a preferred option for sustainable cultivation.

### 3.3. Gel Culture

Gel cultivation is an agricultural technique that uses biodegradable gels as soil or substrate improvers. It is formed by cross-linking cellulose derivatives with citric acid. This hydrogel absorbs and locks in much water through its three-dimensional network structure. It aims to improve the water-holding capacity of water-deficient substrates, thereby enhancing the water availability for plants, reducing the frequency of irrigation, and promoting crop growth. Research shows that cellulose-based biodegradable hydrogels can significantly improve the water-holding performance of sandy soil and perlite substrates [59]. For example, the field water-holding capacity of sandy soil can increase by up to 400%, and it promotes the growth of cucumbers and sweet basil in short-term cultivation. Although the effectiveness of hydrogels may weaken during salt accumulation and long-term wet–dry cycles, their environmentally friendly characteristics and positive effects on plant growth give them application potential in agriculture, but further evaluation of their economic feasibility is needed.

Some scholars have studied the effect of pectin-activated carbon hydrogels (PAC), a composite hydrogel based on passion fruit pectin and activated carbon, on the growth of mung beans in soilless cultivation [60]. The study found that adding activated carbon significantly improved the hydrogel’s microporous structure, water-holding capacity, and mechanical strength. The compressive strength increased from 80.26 to 94.02 MPa. The surface area of the activated-carbon composite hydrogel (28.771 m^2^/g) was much higher than that of the pure pectin hydrogel (15.063 m^2^/g). PAC3 achieved a 100% germination rate within two days, and the seedlings’ root length, stem length, and biomass were better than those of other groups and traditional soil cultivation. Activated carbon enhances the pore structure and stability of the hydrogel through physical effects, promoting the release of water and nutrients and providing an environmentally friendly and efficient material for soilless cultivation, especially suitable for home gardening or rooftop planting.

Wang et al. successfully synthesized a variety of agar-based hydrogels for the cultivation of selenium-enriched vegetables (such as pak choi) [61]. The hydrogel system with the addition of nano-silicon (NS), selenium-chitosan (SeCA), and activated carbon (AC) can significantly extend the selenium-enriched cultivation cycle. The yield of pak choi increased by 191.09% compared with the original agar hydrogel, and the selenium content increased by 10.77%. The three additives have a synergistic effect on plant growth and selenium enrichment. This study provides a new type of substrate for selenium-enriched agriculture in indoor and space environments. It is expected to promote the development of precise and sustainable selenium-fortified cultivation technologies.

**Figure 2:**
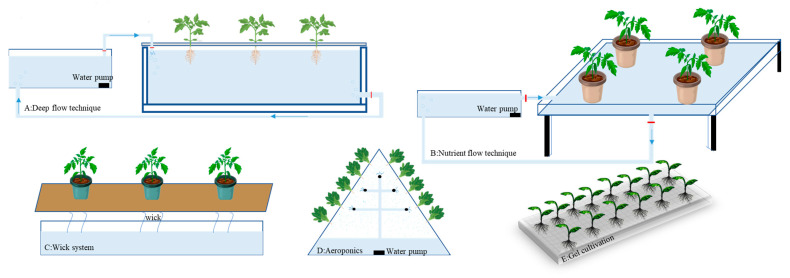
Types of non-solid substrate cultivation.

Dai et al. proposed a new type of soilless cultivation substrate by introducing carbon black into hydroxyethyl cellulose–gellan gum hydrogel beads. This modification significantly improved the substrate’s temperature-regulating ability, pore connectivity, water-holding capacity, and light-shielding effect [62]. The hydrogel beads containing carbon black greatly promoted rapeseed germination and root development under low-temperature conditions. Compared with the substrate without carbon black, the root length increased by 648%, and the number of root tips and branches increased by 637% and 427%, respectively. This substrate efficiently addresses agricultural resource limitations under climate change by optimizing nutrient slow-release, oxygen circulation, and microenvironment stability. It shows the potential to enhance crop growth resilience, especially under low-temperature stress situations. (See Figure 2).

## 4. Nutrient Solutions in Soilless Cultivation

**Figure 3:**
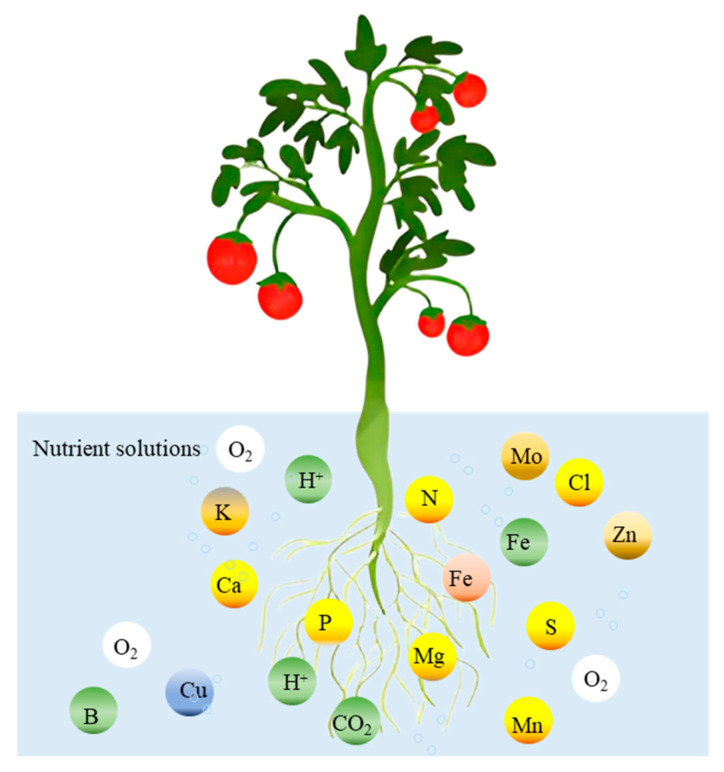
Necessary elements in nutrient solutions of soilless cultivation.

A nutrient solution is prepared by dissolving in water, in specific quantities and proportions, compounds containing various essential nutrients for plant growth and development, along with a small number of auxiliary materials to prolong the effectiveness of certain nutrients (Table 5). The nutrient solution mainly provides nutrients and water for crop growth and development, whether in soilless cultivation with solid or liquid substrates (See Figure 3). The success of soilless cultivation largely depends on whether the formula and concentration of the nutrient solution are appropriate, and whether the nutrient solution management can meet the needs of plants at different growth stages. Climatic conditions, water quality in different regions, and crop species and varieties significantly impact the nutrient solution [7].

Majidi et al. explored the effects of different concentrations of Hoagland nutrient solution (1/4, 1/2, 1, and 2 times) and foliar spraying of sodium silicate (0, 50, 100, and 150 mg/L) on the growth of the medicinal plant bitter gourd through a hydroponic experiment [63]. The study found that the double-strength Hoagland nutrient solution significantly improved the morphological and yield indicators of the plants and shortened the time from flowering to fruit harvest. In contrast, the 1/4-strength solution performed the worst. Sodium silicate at a 100–150 mg/L concentration enhanced root development and promoted nutrient absorption efficiency. It had the best synergistic effect with the double-strength Hoagland nutrient solution, producing the highest chlorophyll a and b contents. However, the carotenoid content was higher in the low-strength Hoagland solution. The high-concentration nutrient solution met the plant’s needs by providing sufficient N, P, and K, while sodium silicate promoted growth by improving the cell wall structure, regulating hormone balance, and enhancing antioxidant capacity.

He et al. investigated the impact of electrical conductivity (EC) in the nutrient solution on the leaf transcriptome and fruit quality of cucumbers in coconut coir cultivation [64]. They found that a medium EC level (5 dS/m) significantly promoted the accumulation of metabolites related to fruit quality by inducing changes in the whole-transcriptome of leaf gene expression, thus optimizing fruit quality. However, a high EC (8 dS/m) activated stress-response genes but inhibited photosynthetic efficiency and secondary metabolite synthesis, leading to metabolic imbalance.

Hershkowitz et al. investigated the effects of increased root-zone phosphorus (P) and nutrient solution EC on the yield and cannabinoid content of medical cannabis through a closed-loop hydroponic experiment [65]. They found that although the P input increased from 15 to 90 mg/L and the EC increased from 2 to 4 mS/cm, there were no significant changes in the dry flower yield, cannabinoid concentration, or plant health. Although the high P input led to a 100% and 70% increase in the phosphorus content in the leaves and flowers, respectively, and the P accumulation in the root-zone solution reached up to 300 mg/L, over-fertilization did not improve the yield or quality but increased the environmental burden. Cannabis was tolerant to high-concentration nutrient solutions, but moderate fertilization was sufficient to meet the growth needs, and over-application was neither economical nor environmentally friendly.

Vought et al. compared the dynamics of micro- and macro-nutrients in lettuce grown in a nutrient film technique system under different EC conditions (EC: 1.2 vs. 1.6 dS/m). They found that increasing the EC did not significantly affect the lettuce biomass or nutrient absorption efficiency. However, it led to significant changes in the nutrient solution composition: persistent calcium (Ca) accumulation. In contrast, the concentrations of potassium (K) and manganese (Mn) decreased. The nitrogen (N) loss rate was 27–40%, and the phosphorus (P) loss was 11–35%. Most of the nutrients, such as N, P, K, and Ca, remained in the solution at a high proportion of 50–80%, and the plant nutrient absorption efficiency was generally low, with the absorption efficiencies of Ca, Mg, and Fe being only 4–6%, 3–4%, and 2–4%, respectively [66].

Behtash et al. explored the role of Zn in alleviating Cd toxicity in hydroponic lettuce [67]. Adding Zn significantly improved the growth parameters and antioxidant enzyme activities of lettuce and reduced the content of oxidative stress markers induced by Cd. A high Zn concentration (10 mg/L) effectively alleviated Cd toxicity in plants by reducing Cd accumulation in leaves and roots, maintaining membrane integrity, and enhancing the antioxidant system. Zn provided a feasible strategy for the safe production of crops in Cd-polluted environments by regulating the Cd:Zn ratio, competing for absorption sites, and reducing the bioavailability of Cd.

A study by Behtash et al. revealed that increasing the concentrations of zinc (5–10 mg/L) and potassium (78–117 mg/L) significantly increased the chlorophyll content, yield, and antioxidant enzyme activities of spinach, while reducing oxidative damage indicators and sodium absorption [68]. However, excessive intake of zinc and potassium can inhibit the absorption of iron, copper, calcium, manganese, and magnesium, leading to a nutritional imbalance. The study recommended adding a combination of 5 mg/L zinc and 78 mg/L potassium to the nutrient solution to optimize the yield, stress resistance, and nutritional quality of spinach while avoiding antagonistic effects on other trace elements.

## 5. Microorganisms in Soilless Cultivation

**Figure 4:**
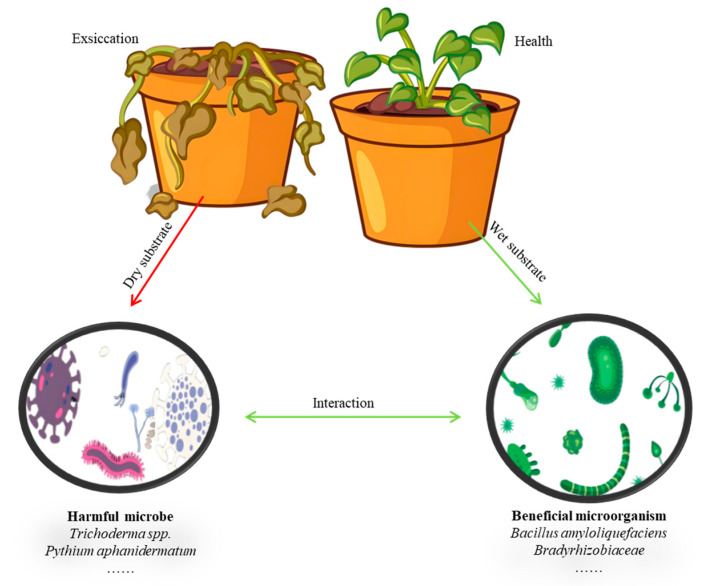
Harmful and beneficial microorganisms in soilless cultivation.

Rhizosphere microorganisms inhabit the plant roots and mainly use the exudates of the roots and easily decomposable dead cells as nutrients. The types of these microorganisms vary depending on the plant species, growth and developmental stages, and the properties of the rhizosphere medium [6]. They can only develop and become active when the substrate has sufficient moisture. If the substrate is too dry, the water film around the rhizosphere medium becomes thinner, which affects the motility of bacteria, the development of fungal and actinomycete hyphae, and the diffusion of nutrients. If the substrate is too wet, it affects the aeration of the rhizosphere, which is unfavorable for aerobic microorganisms. Most microorganisms, such as rhizobia, are nitrogen-fixing and phosphate-solubilizing bacteria that are aerobic. Their intense respiration requires a substrate that is relatively loose, with good structure and aeration. A well-structured rhizosphere substrate is beneficial to the growth and reproduction of microorganisms. Therefore, attention must be paid to the aeration in hydroponics and the water–air ratio in solid substrate cultivation. The crop “rhizosphere-microorganism” system can roughly summarize the interaction among plants, beneficial microorganisms, and harmful microorganisms [69] (See Figure 4).

Selecting specific raw materials for plant growth media and inoculating diverse bacterial communities can independently regulate the structure and diversity of the rhizosphere microbial community in hydroponic lettuce, thereby promoting plant growth. Research has found that *Bacillus* and *Actinomycetes* introduced by green waste compost, as well as *Pseudomonas* introduced by the inoculated bacterial community, are significantly correlated with plant growth [69,70,71]. This study challenges the traditional view that hydroponic systems are sterile environments, emphasizing the importance of microbial diversity for plant health. It also highlights that the rational design of growth media and the optimization of bacterial inoculation strategies are key to improving the sustainability and production efficiency of controlled-environment agriculture.

The organic soilless cultivation system alters the microbial community structure of the tomato rhizosphere, substrate, and fruits, leading to a microbial imbalance, as evidenced by a significant decrease in the abundance of beneficial bacteria, such as *Bradyrhizobiaceae*, *Caulobacteraceae*, and *Streptomycetaceae*, whereas the abundance of the potential pathogen Enterobacteriaceae increases significantly. This imbalance may be related to decreased tomato yield, fruit quality defects, and increased pathogen susceptibility in the organic system [70]. The microbial diversity distribution in the organic system is more uneven, and the functions of the core microbial groups—*Proteobacteria*, *Actinobacteria*, and *Firmicutes*—may be impaired. The enrichment of *Enterobacteriaceae* in fruits may pose a threat to food safety. This finding challenges the perceived inherent advantages of organic agriculture for plant health and emphasizes the need to balance production efficiency and food safety by regulating the microbial community.

Wei et al. compared the rhizosphere microbial communities and metabolites of hydroponic and soil-cultivated Chinese chives. They revealed that hydroponics can regulate specific microorganisms, such as *Rhodanobacter*, *Chujalbacter*, and *Thermomonas*, whose metabolic activities significantly promote the growth and development of Chinese chives [18]. Under hydroponic conditions, microbial diversity is relatively low; however, the dominant microbial groups optimize the rhizosphere microenvironment through nitrogen conversion, denitrification, and detoxification. Simultaneously, metabolomics reveals that carbohydrates and amino acids are down-regulated, whereas phenols, ketones, and amines are up-regulated, enhancing nutrient absorption efficiency and stress resistance. However, hydroponics may weaken the formation of flavor substances because of reduced secondary metabolites.

Research by Van Gerrewey et al. indicates that the rhizosphere microbial diversity of hydroponic lettuce in the Netherlands is only one-fifth of that of soil-cultivated lettuce. However, the abundance of specific functional bacteria increases tenfold. The soilless system may achieve functional enhancement through “microbial streamlining,” but the long-term ecological cost remains controversial [71].

In the soilless floating cultivation system, introducing plant growth-promoting rhizobacteria (PGPR) to replace part of mineral fertilizers can maintain lettuce yield and significantly improve its quality [72,73]. PGPR promote lettuce growth through mechanisms such as nitrogen fixation, phosphorus solubilization, and secretion of plant hormones. Concurrently, PGPR elevate the content of phenolic compounds, flavonoids, vitamin C, and total soluble solids in the leaves, and optimize the absorption of mineral elements such as nitrogen, phosphorus, and calcium. Although PGPR have limited effects on supplementing potassium and magnesium, it demonstrates potential in reducing the dependence on chemical fertilizers, lowering production costs, and reducing the environmental burden, providing a feasible strategy for sustainable hydroponic agriculture. The research suggests that in the future, in-depth exploration of the strain specificity of PGPR, colonization dynamics, and collaborative optimization with cultivation conditions will be needed to promote its large-scale application.

Plocek et al. studied the effects of *Bacillus amyloliquefaciens* and *Trichoderma* spp. on the growth and development of *Brassica rapa* var. chinensis in deep-water culture systems and nutrient film techniques [21]. Trichoderma had a significant negative impact on both hydroponic systems’ physiological indicators, nutrient content, and nutrient use efficiency (NUE) in *Brassica rapa* var. *chinensis*. It is speculated that the excessive growth of its mycelium led to competition with plants for nutrients and oxygen. In contrast, *Bacillus amyloliquefaciens* showed potential as a bio-fertilizer in the nutrient film technique system and notably improved NUE in the deep-water culture system. However, the difference between its effect and the control group was limited, possibly due to the sufficient nutrients in the hydroponic system. The study concludes that Trichoderma at the existing inoculation concentration is not suitable for hydroponic *Brassica rapa* var. *chinensis*, while *Bacillus amyloliquefaciens* can be an optimized choice and the nutrient film technique system performs better overall than the deep-water culture system. In the future, further exploration of microbial inoculation strategies, environmental adaptability, and the influence of organic/inorganic fertilizers is required to improve the sustainability of bio-fertilizers in hydroponics.

Stouvenakers et al. investigated the inhibitory mechanism of water against *Pythium aphanidermatum*, a pathogen causing root rot in lettuce. They found that the microbial community in hydroponic water was the key to disease suppression [74]. Unfiltered hydroponic water significantly inhibited the hyphal growth of the pathogen, although the inhibitory effect disappeared after filtration removed the microorganisms. Lettuce grown in hydroponic water showed significantly reduced disease symptoms after pathogen inoculation, whereas traditional hydroponic nutrient solutions or hydroponic water supplemented with nutrient salts had poorer effects. The microbial diversity in hydroponic water and specific bacterial genera such as *Burkholderiaceae*, *Sphingomonadaceae*, and *Lactobacillus* were closely related to disease suppression. Moreover, the stability of the microbial community showed strong resistance to interference during pathogen invasion. Altering the physical and chemical properties of hydroponic water disrupted its microbial composition, weakening the antibacterial effect.

In aquaponic systems, microbial strains screened and isolated through high-throughput sequencing technology effectively inhibited lettuce root rot and damping-off caused by *Pythium* pathogens. The study found that the control effects of these strains were better than those of traditional fungicides and registered biological control agents, and they could maintain plant health while reducing diseases. For the first time, the study verified the potential of aquaponic systems as a source of novel biological control agents. It proposed a targeted isolation strategy based on high-throughput sequencing, providing new ideas for developing sustainable disease management programs adapted to soilless cultivation environments [75]. 

## 6. Application of Soilless Culture Technology

Soilless cultivation technology has been widely used in the production of horticultural crops and economic crops, such as vegetables, flowers, and fruit trees, especially in greenhouses and other facilities. It has become one of the indispensable technical means, which has better solved the continuous cropping obstacles and quality problems of horticultural crops cultivated in facilities. Soilless cultivation technology also plays an important role in sightseeing agricultural parks and space agriculture.

The application of soilless cultivation technology in modern greenhouse industry is mainly reflected in the replacement of soil by inert substrates such as rock wool and coconut bran or hydroponic systems such as nutrient liquid film and aeroponics, combined with automatic irrigation and fertilization equipment to accurately regulate nutrient solution components (EC, pH, ion concentration) and irrigation strategies to achieve crop root zone environment optimization. Through the closed system for recycling disinfected drainage, the efficiency of water and fertilizer utilization is significantly improved, and environmental pollution is reduced. At the same time, salt regulation and environmental matrix development (such as biochar) are used to improve the quality and yield of agricultural products while avoiding soil-borne diseases, and expand to non-cultivated areas such as saline-alkali land, becoming an efficient and sustainable facility agriculture core solution [28].

The application of soilless cultivation technology in ornamental plants is primarily reflected in the replacement of soil with a solid matrix or a hydroponic system, combined with automatic irrigation and fertilization to precisely regulate the composition of the nutrient solution, thereby optimizing the root-zone environment. A closed system is used to recycle the effluent to improve resource efficiency, and environmentally friendly substrates (such as olive seed waste, papermaking waste, and vermicompost instead of peat) are developed to reduce the ecological footprint. This technology has significantly improved the yield and quality of cut flowers (such as roses, lilies, and tulips) and potted plants (including geraniums and cyclamens) by increasing the length of flower stems and advancing the flowering period. At the same time, it has promoted the simple hydroponic system for use in home gardening, expanding the application scenarios [76].

The fish–vegetable symbiotic system belongs to the branch of soilless culture. María Carmen Piñero used tilapia aquaculture wastewater, converted into a nutrient solution (containing a low concentration of nitrate) by nitrifying bacteria, and combined it with a coconut bran matrix to cultivate lettuce, thereby realizing circular production of aquaculture and planting. In this study, selenium (16 μmol·L^−1^) was sprayed on the leaves to alleviate low nitrogen stress: selenium increased nitrate absorption efficiency by 56%, brought the nitrate concentration of lettuce close to the conventional cultivation level, reduced lipid peroxidation damage by 16%, regulated sugar metabolism (reduced fructose and inositol content), and increased antioxidant enzyme (catalase) activity by 28%. This technology integrates wastewater recycling and selenium bioaugmentation, which not only reduces the use of synthetic fertilizers and water pollution but also improves the nutritional quality of lettuce [77].

Soilless culture can also be used in scientific research. Nikolaos Tzortzakis discussed the interactive effects of different nitrogen levels (75, 150, 300 mg L^−1^), excessive copper stress (100 μM vs. 5 μM control), and zinc foliar spraying (1.74 mM vs. none) on the growth, physiology, and metabolism of *Sideritis cypria* under hydroponic conditions. In a copper-contaminated hydroponic system, the use of medium- and low-nitrogen nutrient solutions, combined with zinc foliar spraying, was found to optimize the growth, quality (secondary metabolites), and stress tolerance of Clematis in Cyprus, providing a feasible strategy for the safe cultivation of medicinal plants [78].

Haozhen Nie et al. systematically discussed the response mechanism of plants in a space microgravity environment and its significance for the development of space agriculture. This paper emphasized the key technologies of space agriculture, including bioregenerative life support systems, soilless cultivation, LED light optimization, and breeding of space-appropriate crops. It highlighted that space mutation breeding can significantly enhance the crop mutation rate and stress resistance. The review looks forward to future research directions, including the integration of synthetic biology, epigenetics, and in situ resource utilization to support sustainable food and life support systems for long-term human space exploration [79].

Some scholars have also done relevant research on soilless cultivation and AI applications [80,81]. However, high costs, technical complexity, energy demand, data security risks, and a lack of policies have seriously restricted adoption and may exacerbate agricultural inequality. In the future, low-cost technology development, policy support (such as subsidies and regulatory updates), farmer training, and cross-disciplinary cooperation will be necessary to achieve a technologically inclusive and environmentally friendly agricultural transformation.

## 7. New Path for Soilless Cultivation

The advancement of soilless cultivation technology has enabled humans to control environmental conditions during different crop growth stages precisely. As a result, agricultural production may be freed from the constraints of natural conditions and develop in directions such as precision, spatial utilization, mechanization, intelligence, and industrialization, thereby meeting human needs and significantly improving crop yield and quality. The development and utilization of new substrates, the optimization of substrate physical and chemical properties, and the interaction between soilless cultivation and microorganisms are expected to become new paths and future research hotspots.

As a key innovation in modern agricultural technology, soilless cultivation overcomes the limitations of traditional soil-based planting by precisely regulating the crop growth environment, providing an important path for achieving efficient and sustainable agricultural production. In the future, it will be necessary to develop new substrates derived from agricultural waste, nanomaterials, and degradable hydrogels to reduce the environmental burden, integrate AI, big data, and sensor technologies to achieve intelligent and precise environmental control and personalized nutrition plan design, construct functional microbial communities through synthetic biology, optimize microbiome engineering to enhance system disease resistance, nutrient cycling efficiency, and crop quality, and integrate the advantages of multiple disciplines such as materials science, environmental engineering, and biotechnology to promote the expansion of soilless cultivation into areas such as low-carbon agriculture.

**Figure 5:**
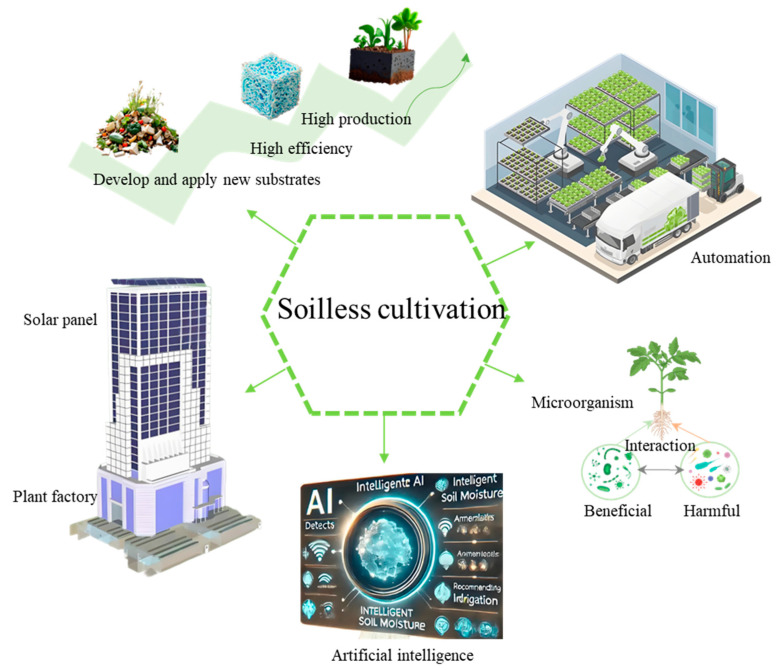
The integration of soilless cultivation with other technologies.

Soilless cultivation technology provides a revolutionary solution to address food security, resource shortages, and environmental degradation. Although breakthroughs are still needed in areas such as substrate recycling, cost control, and the mechanisms of plant–microbe interactions, it holds great innovation potential in terms of intelligence, sustainability, and multifunctionality. In the future, through multidisciplinary cross-fertilization and technological integration, soilless cultivation is expected to become the main driver of global agricultural transformation, helping humans move towards a new era of efficient, green, and circular agricultural production. (See Figure 5).

## Figures and Tables

**Table 1:** Characteristics of some current soilless cultivation solid substrates.

Type	Name	Origin	Advantage	Disadvantage
Inorganic	Sand	Rivers, seas, lake shores and deserts, and other places.	Affordable prices and strong drainage.	Low carbon, nitrogen content, and water holding capacity. No salt exchange capacity. Poor water retention capacity
	Coal cinder	Coal-fired boilers (such as industrial boilers, heating boilers) waste residue after combustion.	Good aeration and moderate bulk density are beneficial for fixing crop roots.	Slightly alkaline, poor water retention and absorption capacity, uneven texture, and small heat capacity.
	Vermiculite	Porous particles formed by natural mica minerals after high temperature expansion treatment.	Good gas permeability, water absorption, and water holding capacity.	When used multiple times, its physical properties will change.
	Perlite	The porous white particles formed by high temperature (about 1000 °C) calcination and expansion of natural volcanic rocks.	Stable, sturdy, lightweight, clean and sterile, with good drainage and gas permeability.	Poor water and fertilizer retention.
	Rockwool	A fibrous inorganic material made from natural ores such as basalt or diabase by high temperature (about 1600 °C) melting and centrifugal spinning.	Chemical stability, excellent physical properties, pH stability, and no pathogenic bacteria carried.	Extremely difficult to decompose in soil after abandonment.
Organic	Peat	Semi-decomposed organic matter formed by incomplete decomposition of plant residues in swamps or wetlands for thousands of years under anaerobic conditions.	Lightweight texture, excellent water retention, good breathability, high nutritional content.	Non-renewable natural resources, long-term use will lead to resource depletion.
	Rice hull	Grain shell removed during rice processing.	Good permeability.	The water and fertilizer retention performance is ordinary, with low nutrient content and high pH.
	Sawdust	Sawdust or particle waste from wood processing (e.g., sawmills, furniture factories).	Rich sources, light bulk density, good water absorption and retention properties.	The carbon to nitrogen ratio is too high, with mixed bacteria and pathogenic microorganisms.
	Bagasse	The remaining fibrous residue after sugarcane juice in sugar industry.	The by-products of the sugar industry come from a wide range of sources.	Fresh sugarcane bagasse has a high carbon to nitrogen ratio and cannot be used directly.
	Coir	Fine powder produced during the processing of coconut shell fiber.	Long fiber, loose and porous, with good water retention and gas permeability.	Need to develop and utilize molded products.

**Table 2:** Economic efficiency indicators of different cultivation methods.

Index	Soil Culture	Soilless Culture			
		Substrate	Hydroponic	Aeroponics	Gel Culture
Investment costs	Low	Medium	High	High	Highest
Technical difficulty	Low	Medium	High	Very high	Medium
Management and maintenance workload	High	Medium	Medium	High	Low
Yield and growth rate	Low	Medium	High	Very high	high
Energy consumption (water, electricity, etc.)	High	Medium	High	Very High	Low
Disease transmission risk	Very high	Medium	High	Very High	Low
Economic Benefit	Low	Medium	High	High	Medium

**Table 3:** Physical properties of several common substrates [34].

Substrates	Bulk Density (g·cm^−3^)	Total Porosity (%)	Aeration Porosity (%)	Water Holding Porosity (%)	Air-Water Ratio
Soil	1.10	66.00	21.00	45.00	0.47
Sand	1.49	30.50	29.50	1.00	29.50
Vermiculite	0.13	95.00	30.00	65.00	0.46
Perlite	0.16	93.00	53.00	40.00	1.33
Rockwool	0.11	96.00	2.00	94.00	0.02
Peat	0.21	84.40	7.10	77.30	0.09
Vinegar residue	0.21	84.52	46.40	38.10	1.22
Sawdust	0.19	78.30	34.50	43.80	0.79
Carbonized rice husk	0.15	82.50	57.50	25.00	2.30

**Table 4:** Analysis of chemical properties of different cultivation methods [34].

Index	Traditional Soil	Substrate	Hydroponic	Aeroponics	Gel Culture
Chemical stability	Low	Medium	Low	Very Low	Very High
Nutrient control precision	Low	Medium	High	Very High	High
pH buffering capacity	Very High	Medium-High	Low	Very Low	Medium
Cation Exchange Capacity (CEC)	High	Variable **	None	None	Controlled
Salinity control	Low	Medium	High	Very High	Medium-High
Synthesized rating	Low	Medium	High	High	Very High

** Substrate CEC: Peat (high), rockwool (none), cocoir (medium).

**Table 5:** Nutrient elements necessary for plants.

Nutrient Element		Available Forms of Plants	Compound of Fertilizer
Macro elements	C	CO_2_	-
	O	O_2_, H_2_O	-
	H	H_2_O	-
	N	${\mathrm{NO}}_{3}^{-}$, ${\mathrm{NH}}_{4}^{+}$	NH_4_NO_3_, (NH_4_)_2_SO_4_, CO(NH_2_)_2_, Ca(NO_3_)_2_·4H_2_O
	K	K^+^	KNO_3_, K_2_SO_4_, KCl
	Ca	Ca^2+^	CaSO_4_·2H_2_O, CaCl_2_
	Mg	Mg^2+^	MgSO_4_·7H_2_O
	P	H_2_${\mathrm{PO}}_{4}^{-}$, ${\mathrm{HPO}}_{4}^{2-}$	Ca(H_2_PO_4_)_2_, Ca(H_2_PO_4_)_2_·H_2_O, CaSO_4_·2H_2_O
	S	${\mathrm{SO}}_{4}^{2-}$	KH_2_PO_4_, NH_4_H_2_PO_4_, (NH_4_)_2_HPO_4_
Micro elements	Cl	Cl^−^	FeCl_3_·6H_2_O
	Fe	Fe^3+^, Fe^2+^	FeSO_4_·7H_2_O
	Mn	Mn^2+^	MnSO_4_·4H_2_O or MnSO_4_·H_2_O
	B	${\mathrm{BO}}_{3}^{3-}$, B_4_$O_{3}^{2-}$	H_3_BO_3_, Na_2_B_4_O_7_·10H_2_O
	Zn	Zn^2+^	ZnSO_4_·7H_2_O, ZnCl_2_
	Cu	Cu^2+^, Cu^+^	CuSO_4_·5H_2_O, CuCl_2_·2H_2_O
	Mo	${\mathrm{MoO}}_{4}^{2-}$	(NH_4_)_6_Mo_7_O_24_·4H_2_O
